# Reimagining community engagement in TB elimination: a perspective from the field

**DOI:** 10.5588/ijtldopen.24.0578

**Published:** 2025-01-01

**Authors:** A. Ashesh, C. Mehra, V. Madan, J. Furin

**Affiliations:** ^1^ Survivors Against TB, New Delhi, India;; ^2^ Harvard Medical School, Department of Global Health and Social Medicine, Boston, MA, USA.

**Keywords:** tuberculosis, power-sharing, community-centered, person-centred

## Abstract

The role of community engagement (CE) in TB policy, care provision and research has been recognized as important, but most models of CE put communities at the periphery of these activities. In this Editorial, written by TB-impacted community members and care providers, we explore the meaning of CE and current barriers to its implementation. We present a model of CE that places community members at the center of TB policy, care provision and research. The article concludes with advocacy points, including the need for sufficient funding for CE and genuine power-sharing between TB-affected communities and the people and programs aiming to serve them.

Community engagement (CE) is a term that has become increasingly common in TB policy and discourse. However, there are a wide range of interpretations as to its meaning and impact on the global struggle to end TB.^1^ For example, it was recognized as far back as the 1950s when National Institutes of TB were established in India ‘based on public health principles and community-based strategies’.^2^ However, to date, CE generally runs the gamut from being tokenistic to transactional,^3^ and is often treated as a box to tick on the checklist, or as a necessary means to an end, such as to increase the uptake of services.^4^ The problem with both these approaches is that the community is not viewed as inherently valuable or an equal partner in co-producing TB policy and care. The meaningful participation of communities as co-creators in TB policy and care enhances the health system's capacity to provide care and provides person-centred understanding of health needs and barriers to accessing care.^5^ Here, we aim to highlight gaps in the current CE paradigm. Further, we identify the barriers to effective CE and articulate the core principles of meaningful engagement. We conclude with a proposed reimagined framework for CE in TB – our aim being to re-center meaningful engagement of the community in TB policy and practice.

## Current barriers to community engagement in TB

Although the term ‘community’ has multiple definitions across cultures and contexts,^6^ and is wide enough to include practitioners and all stakeholders in the TB care process,^7^ we are using the word in a specific sense: as an impacted community including but not limited to TB affected individuals, TB survivors, their close contacts and civil society organizations. We have focused on these stakeholders because they should be at the centre of CE but are too often marginalised.

As authors, we have experienced multiple barriers to CE in TB policy and practice. In public health, there is no universal or concrete definition for CE, and different stakeholders have different views on engaging communities. This variability or diversity is not in itself an issue – indeed, context-specific definitions of CE, co-developed by communities, are more effective in addressing the specific health needs and access barriers. However, in our experience at the country level, definitions of CE may be created from the top down with only limited involvement of communities. Further, without at least a threshold definition, we lack adequate metrics to assess CE efforts and determine whether meaningful engagement has occurred. At a societal level, TB-related stigma^8^ often deters individuals within a community from engaging in advocacy efforts. At a systemic level, in the absence of institutionalized legal and policy recognition of CE, engagement efforts remain fragmented.^9^ Although the rhetoric for CE is ubiquitous, political will and financial resources to enable meaningful CE are lacking.^10^ Capacity-building in the context of CE often tends to be one-way, with national TB programs aiming to build capacity for communities. Training for communities on TB science and policy is important, but the approach to capacity building needs to be two-way, with national TB programs learning from communities on how best to engage.^11^ Moreover, current CE approaches remain heavily centered on enhancing service delivery,^12^ but meaningful CE in decision-making is lacking. The general trend in this regard, barring a few exceptions, is marginal representation (i.e., one or two people) of communities on subnational, national and international decision-making fora. This is deeply problematic, given that communities are equal partners and architects in designing TB care and policy interventions. Instead, their role is largely reduced to being an implementing group (often unpaid) for the policymakers and service providers in TB care.^13^

## Proposed model for re-centering CE in TB

**Figure 1.**
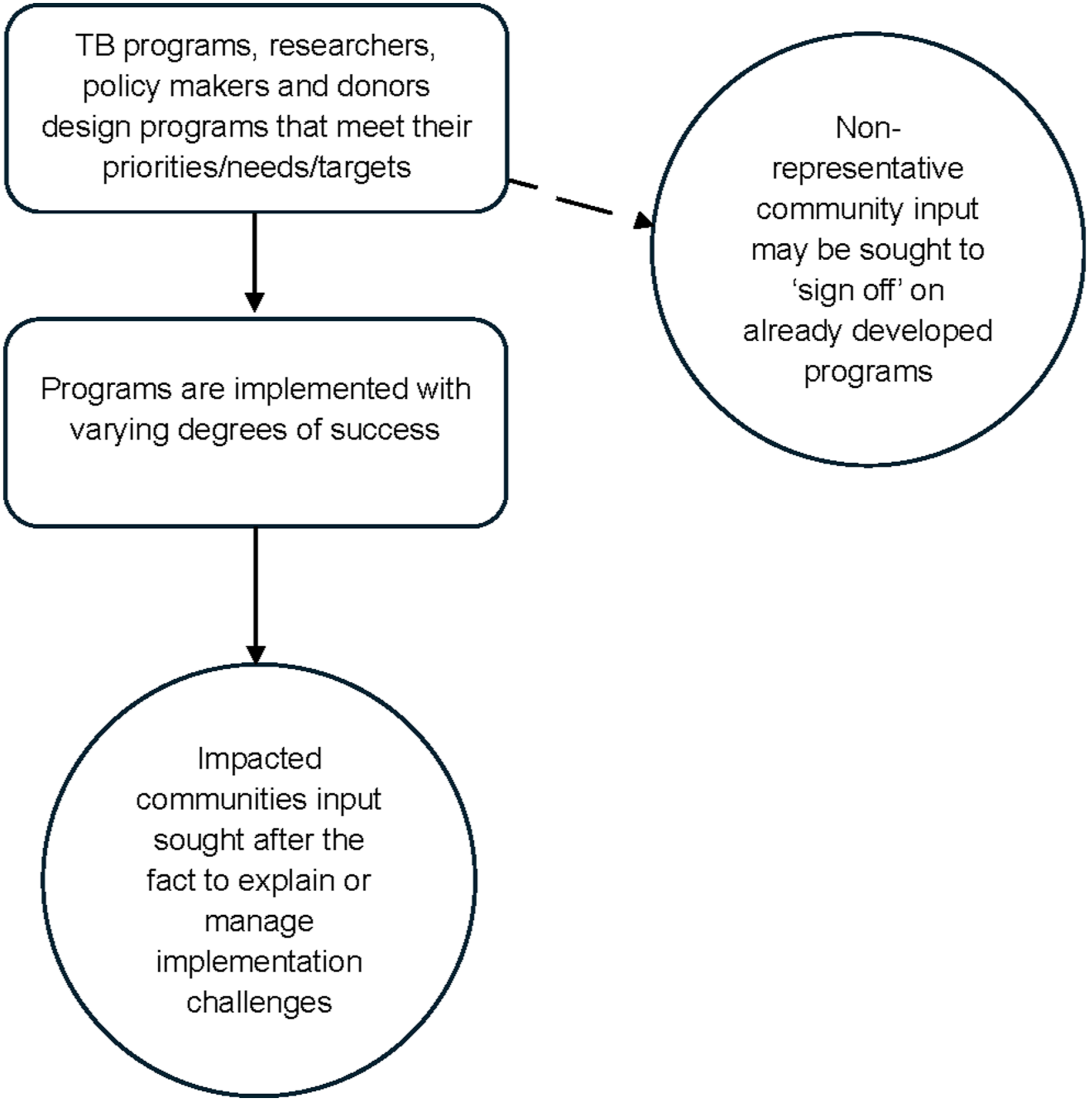
Current models of community engagement in TB.

**Figure 2.**
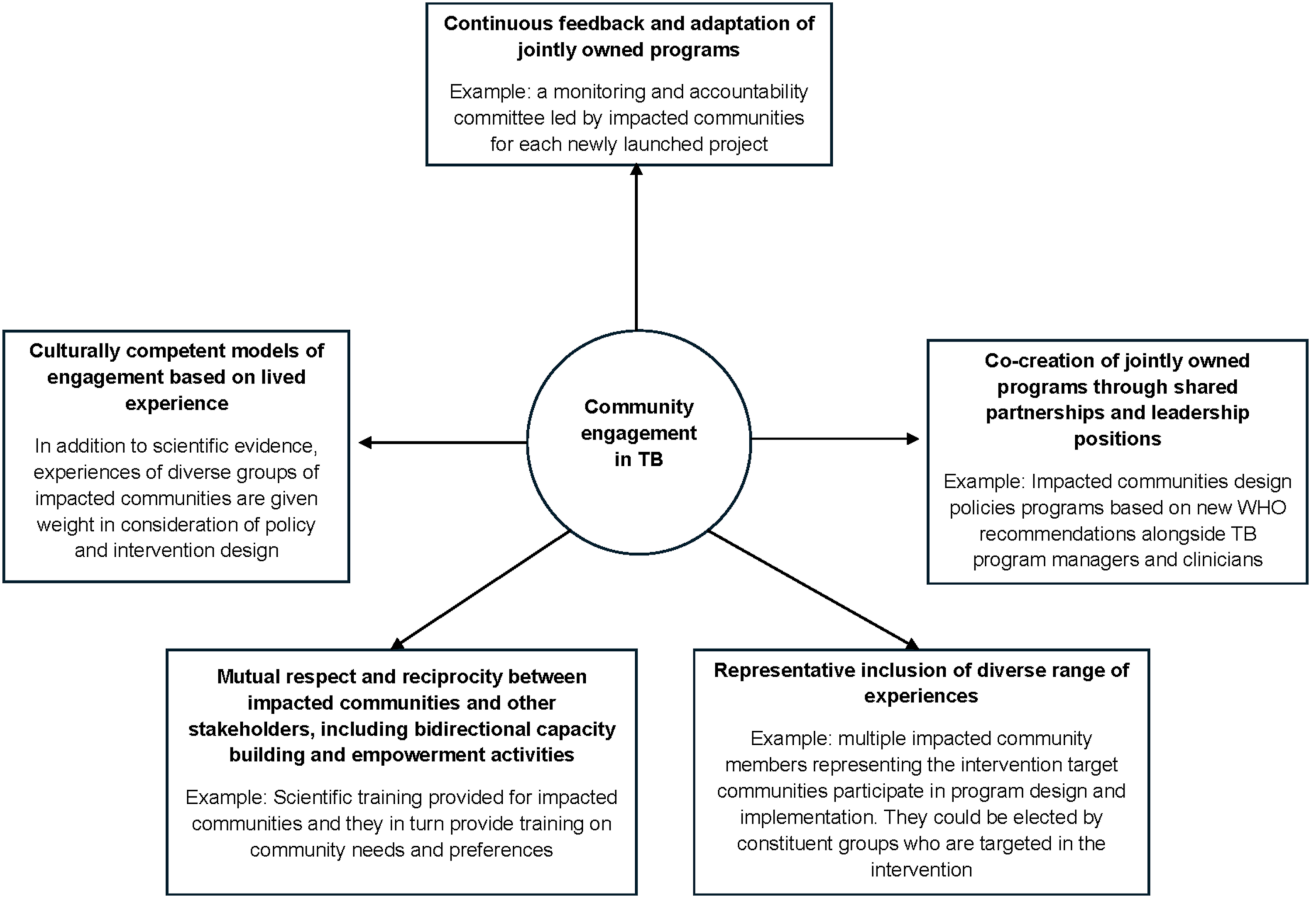
Reimagining community engagement in TB.

Current models of CE in TB tend to place it (when it does occur) at the periphery of program planning (Figure 1). In this model, service delivery, policy and research are largely developed by other stakeholders who then reach out to impacted communities either for ‘sign off’, validation (from a small number of non-representative community members about the planned activities) or to explain/remediate activities that did not yield the desired results. These peripheral models of engagement are not consistent with the authentic interactions that need to happen in TB service delivery and research. In a reimagined model, CE would be central to all planned TB services, policies, and research programs. Impacted communities would provide valued input as ‘co-producers,^14^ given their specialized knowledge at all levels of planning (Figure 2). Representatives across the diverse range of community experiences would hold leadership positions and co-create/co-own the jointly developed activities. All stakeholders would engage in capacity-building efforts whereby their expertise would be shared with one another. Impacted communities would have a forum to share their expertise in providing culturally informed models of care/research based on their lived experience, and this proficiency would be given equal weight to the medical and scientific TB comprehension possessed by other stakeholders. This mutual respect would lead to ongoing feedback and assessment of policy development, program design, and planned activities, with joint accountability for overcoming challenges and propagating successes.

As well-intentioned as CE efforts are in TB programs, they are currently limited by a top-down paradigm with the impacted community at the margins. For TB care to be truly person-centered we need meaningful CE that places communities at the center as equal partners. Co-creating with impacted communities at all levels – including care interventions and policy – requires reimagining what it means to engage. Stakeholders, especially national TB programs, need to evolve in line with this reimagined vision. We are aware that this vision requires substantive political and financial investment, but only in this way can CE move from being tokenistic to transformational.
